# Supplementary material for: A Global Screen for Assembly State Changes of the Mitotic Proteome by SEC-SWATH-MS
Source: Cell Syst. 2020 Feb 26;10(2):133–155.e6. doi: 10.1016/j.cels.2020.01.001 (PMC7042714; doi:10.1016/j.cels.2020.01.001)

Q96F07 | CYFP2_HUMAN | CYFIP2 KIAA1168 PIR121
Monomer MW [kDa]: 148.399 Monomer expected elution fraction: 37

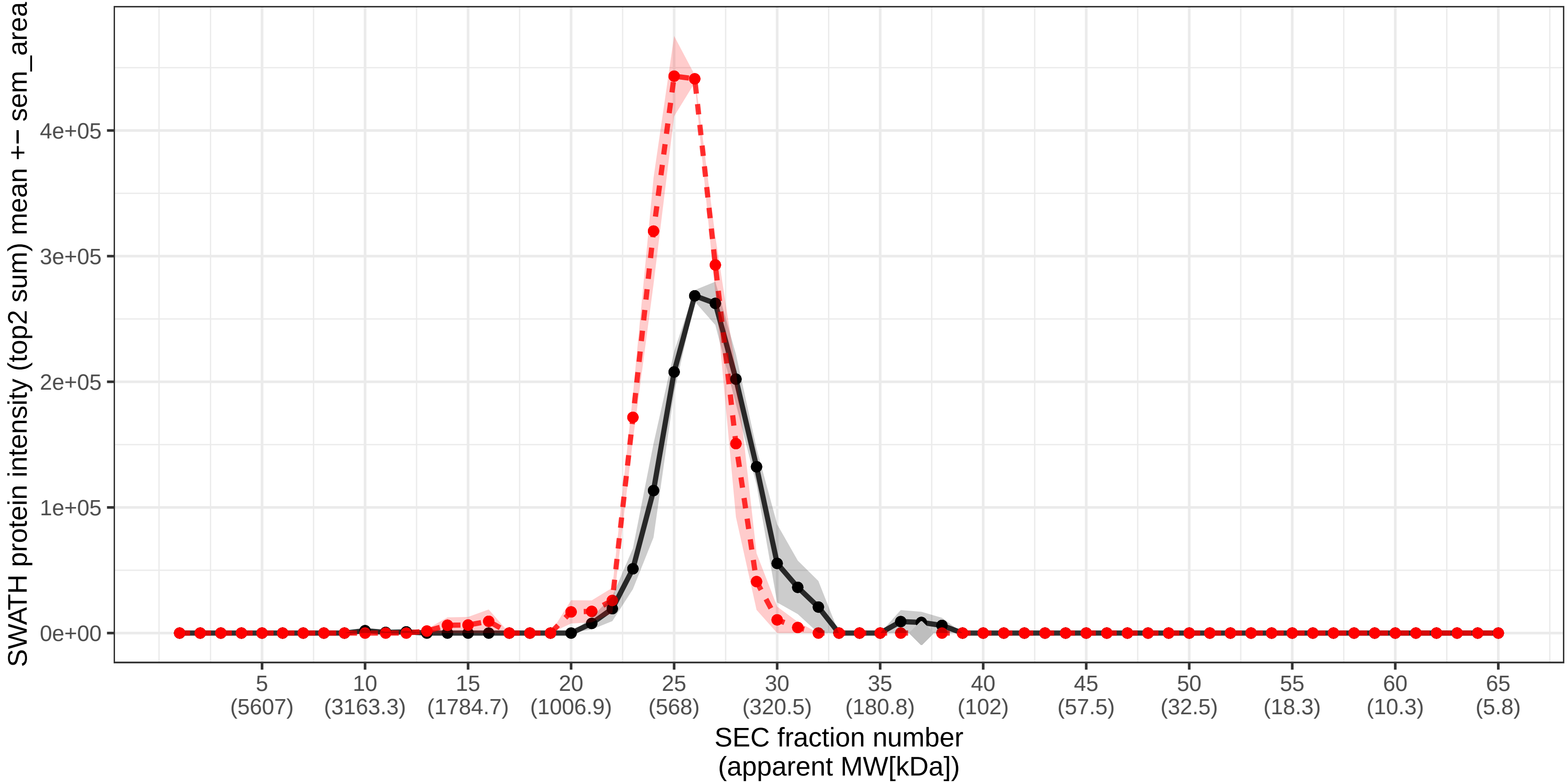

Supplement: Data S1. SEC-SWATH-MS Protein Chromatograms, Related to Figure 1 [file mmc6.zip › SECchrom_Q96F07_CYFP2_HUMAN_CYFIP2_KIAA1168_PIR121.pdf]
